# Mixed mood states and emotion-related urgency in bipolar spectrum disorders: a call for greater investigation

**DOI:** 10.1186/s40345-019-0171-y

**Published:** 2020-03-05

**Authors:** Jennie A. Jaggers, June Gruber

**Affiliations:** Department of Psychology and Neuroscience, University of Colorado Boulder, 345 UCB Muenzinger D321C, Boulder, CO 80309-0345 USA

Bipolar disorder (BD) is a complex and serious psychiatric disorder characterized by mood dysregulation difficulties (American Psychiatric Association 2013; Gruber 2011; Phillips et al. 2008; Johnson et al. 2007; Swann et al. 2013). BD is also associated with a high rate of suicide (Merikangas et al. 2011) and functioning difficulties (Murray and Lopez 1996; Sanchez-Moreno et al. 2009). Moreover, BD patients are at elevated risk for suicide (Koukopoulos et al. 2007) and exhibit poorer responses to treatment interventions (González-Pinto et al. 2010) during mixed mood states (Swann et al. 2013). This underscores the need to better understand psychologically-relevant processes that may contribute to the etiology and course of BD, particularly during mixed mood states.

This brief letter suggests the importance of considering impulsivity during mixed states. *Emotion*-*related urgency* (hereto referred as ERU) is defined as a tendency to act rashly in the context of extreme emotions, including trouble inhibiting impulses and risk-taking behaviors (e.g., Cyders and Smith 2008). ERU has been studied in the context of extreme negative and positive emotions (e.g., Carver et al. 2013; Johnson et al. 2018). For example, *negative urgency* (defined as the tendency to behave rashly during extreme negative emotions; e.g., Whiteside and Lynam 2001) is associated with increased impulsivity during extreme negative mood states, and has been linked to hostile verbal and physical behaviors (Johnson and Carver 2016), compulsive spending (Lejoyeux et al. 2000) and tobacco cravings (Anestis et al. 2007). By contrast, *positive urgency* (defined as the tendency to behave rashly during extreme positive emotions; Cyders 2014) is associated with increased risk-taking and sensation seeking, including excessive drinking (Cooper et al. 1995; Cyders et al. 2009), sexual promiscuity (Johnson and Carver 2016), and aggression (e.g., Cyders and Smith 2008).

Both negative and positive ERU have been implicated in BD (Giovanelli et al. 2013; Johnson et al. 2018), suggesting it may be an important factor underlying risk and functioning difficulties. However, one important gap in these findings is the lack of data on ERU during mixed-states in BD that include, in part, co-occurring negative and positive affective states. Given elevated risk of suicidality and harmful behaviors that co-occur during mixed mood states (e.g., Nordentoft et al. 2011) and the high percentage of mixed mood episodes occurring among BD individuals (González-Pinto et al. 2010; Swann et al. 2013), greater research prioritization is warranted. Yet, we know of no research to date specifically examining ERU in the context of mixed mood states in BD or across mood disorders.

We suggest increased investigation into transdiagnostic presentations and clinical implications of ERU. Indeed, recent recognition of mixed features also occurring in the context of major depressive disorders underscores the important transdiagnostic utility of ERU across mood disorders. We suggest three key areas for future research. First, it will be important to develop well-validated assessment tools to measure mixed-mood ERU across self-report and clinician-rated instrument domains. Second, it will be important to carefully assess the maladaptive behaviors that may predict, and result from, ERU during mixed states and differentiate those from more general risk-taking behavior. Third, future research should carefully consider lifespan approaches that consider ERU in the context of BD onset, risk and recurrence at critical developmental junctures. We believe the time is ripe to call greater attention to examining risky and potentially fatal consequences of impulsivity during strong and co-occurring negative and positive emotion states.

## Data Availability

Not applicable.
